# Supplementary figures and images for: Detection of antibiotic-resistant bacteria endowed with antimicrobial activity from a freshwater lake and their phylogenetic affiliation
Source: PeerJ. 2016 Jun 9;4:e2103. doi: 10.7717/peerj.2103 (PMC4906672; doi:10.7717/peerj.2103)

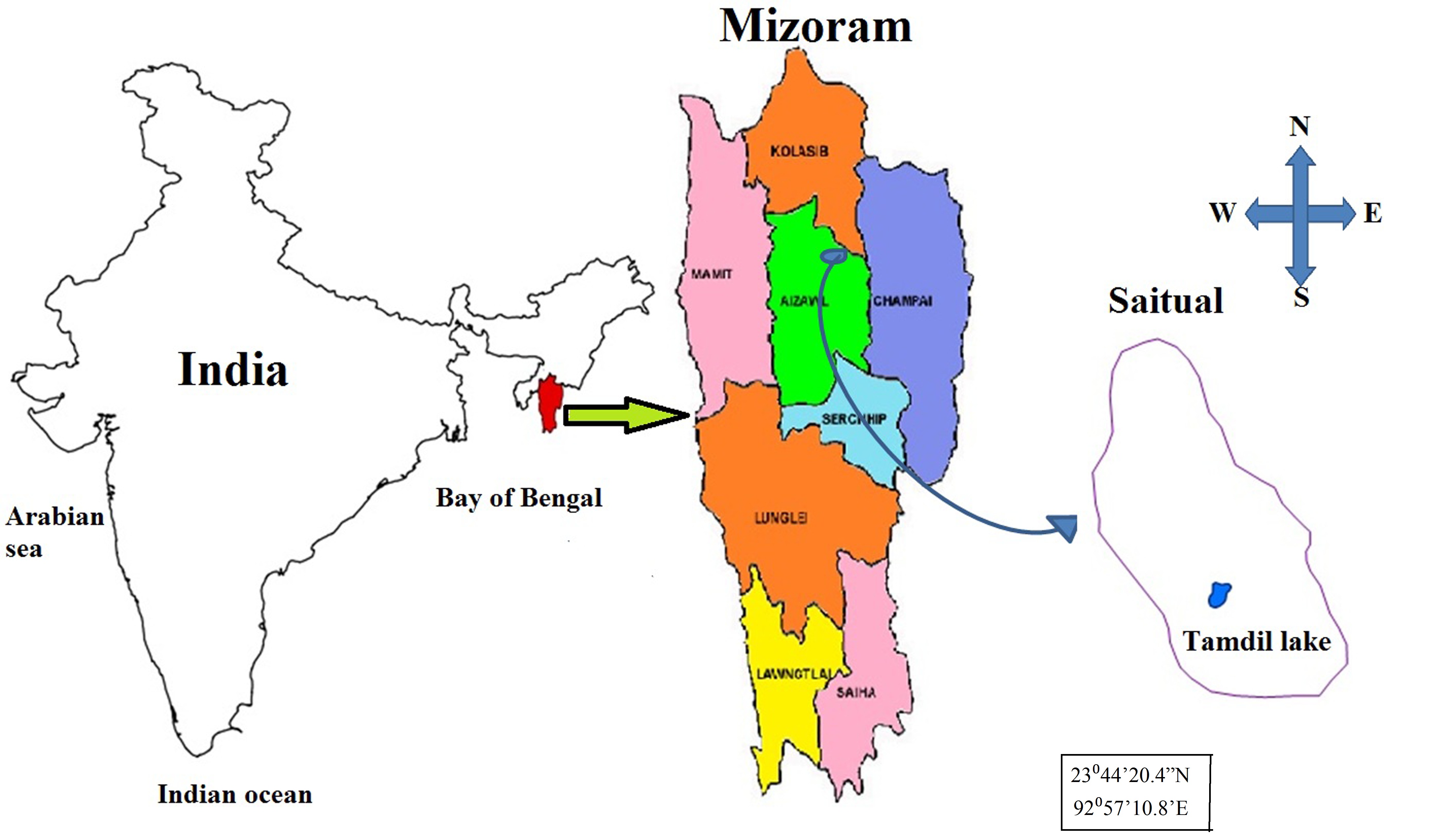

Supplement: Figure S1 [file peerj-04-2103-s001.jpg]

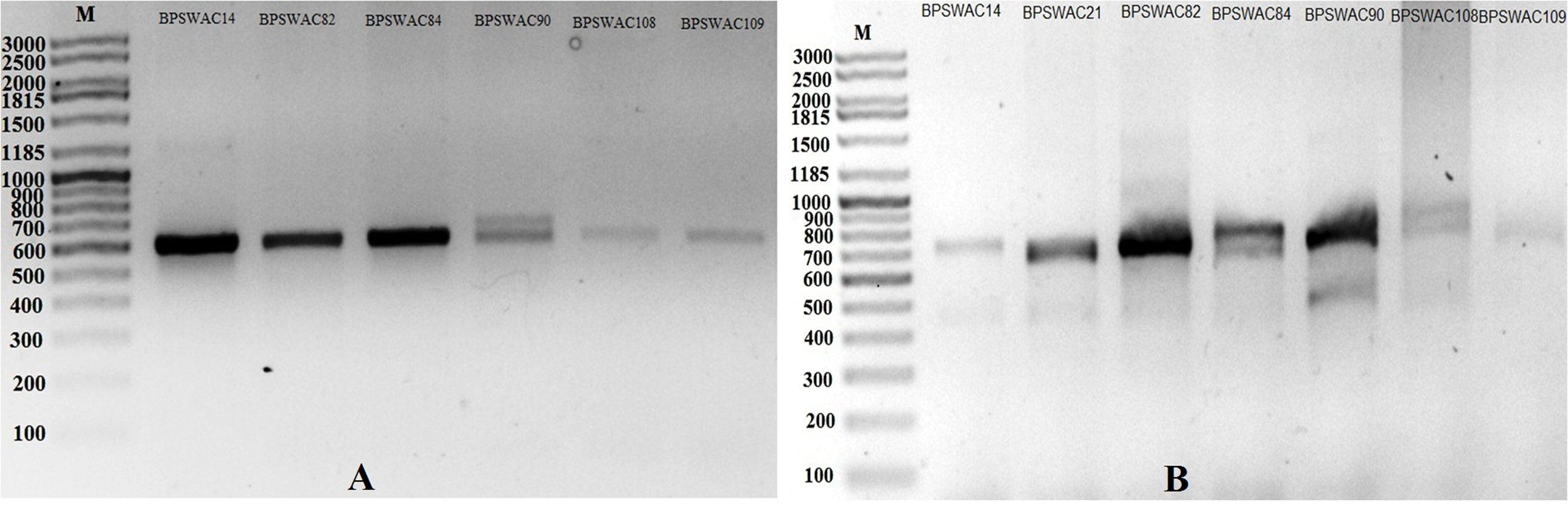

Supplement: Figure S2 [file peerj-04-2103-s002.jpg]
